# Emergency complications during dermatological, surgical, or cosmetic procedures: A cross‐sectional study among dermatologists

**DOI:** 10.1111/jocd.16479

**Published:** 2024-07-20

**Authors:** Hilal Kaya Erdogan, Melisa Sahin Tekin, Esra Agaoglu, Sema Sanal Bas, Ersoy Acer, Zeynep Nurhan Saracoglu, Muzaffer Bilgin

**Affiliations:** ^1^ Department of Dermatology, Faculty of Medicine Eskişehir Osmangazi University Eskişehir Turkey; ^2^ Department of Internal Medicine, Faculty of Medicine Eskişehir Osmangazi University Eskişehir Turkey; ^3^ Department of Anesthesiology and Reanimation, Faculty of Medicine Eskişehir Osmangazi University Eskişehir Turkey; ^4^ Department of Biostatistics, Faculty of Medicine Eskişehir Osmangazi University Eskişehir Turkey

**Keywords:** complication, cosmetic, dermatological surgery, emergency, vasovagal syncope

## Abstract

**Background:**

Medical emergency complications may occur during dermatological, surgical, and cosmetic procedures.

**Aims:**

This study aimed to investigate the frequency of dermatologists who experienced emergency complications as well as their level of knowledge regarding emergencies and basic life support.

**Methods:**

The cross‐sectional descriptive study was conducted online among 240 dermatologists to whom a questionnaire was sent via email and a closed social media group. The survey instrument asked about emergency complications during dermatological, surgical, or cosmetic procedures and the dermatologists' level of knowledge regarding emergencies and basic life support.

**Results:**

Among the dermatologists, 53% reported emergency complications during dermatological and surgical procedures and 43.2% during cosmetic procedures. The most common complications were vasovagal syncope, hypotension/bleeding, and seizures. Emergency complications were more common among specialists, those with more than 15 years of professional experience, those working in their private clinics, and those performing an average of 10–50 dermatological/surgical procedures per week and fewer than 10 cosmetic procedures per week (*p* < 0.05). The knowledge level of dermatologists was highest among residents, dermatologists with 0–4 years of professional experience, those working in university hospitals, and those who had both theoretical and practical training in basic life support.

**Conclusions:**

This study shows a relatively high frequency of dermatologists who experienced emergency complications during dermatological, surgical, or cosmetic procedures. Although these complications seem to be common; most of them are mild, self‐limiting, and not life‐threatening. Nevertheless, dermatologists should be competent and prepared to intervene in medical emergencies in daily practice.

## 1 INTRODUCTION

Medical emergencies are acute, life‐threatening conditions that require immediate intervention. With surgical and cosmetic procedures under local anesthesia becoming increasingly popular in daily dermatology practice, conditions requiring emergency intervention during dermatological, surgical, or cosmetic procedures occur more frequently such as vasovagal syncope, anaphylaxis, hyperventilation syndrome, and cardiac arrest. Early recognition of these emergencies and immediate intervention can save lives, so dermatologists should be competent and prepared to intervene in emergencies, yet healthcare providers, including dermatologists, sometimes do not feel competent to manage emergency conditions.^1^, ^2^ To address this problem, it is important to determine the frequency and characteristics of medical emergencies in dermatological practice as well as the knowledge and skill levels of dermatologists regarding emergencies and basic life support.

Dermatological surgery seems to be safe, and emergency complications associated with these procedures have rarely been described in the literature.^2^, ^3^, ^4^ To the best of our knowledge, no study has evaluated emergency complications in cosmetic procedures and dermatologists' level of knowledge regarding emergencies and basic life support. This study aimed to determine the frequency of dermatologists who experienced emergency complications throughout their professional lives during dermatological, surgical, and cosmetic procedures as well as dermatologists' knowledge level of emergencies and basic life support.

## 2 MATERIALS AND METHODS

This cross‐sectional descriptive study was conducted between December 1, 2022 and January 31, 2023. A web‐based, 33‐item questionnaire was prepared in Google Forms and sent to dermatologists via email and through a closed social media group of the Turkish Society of Dermatology. The local ethics committee approved the study protocol, and the participants provided informed consent online.

The first part of the questionnaire captured the dermatologists' sociodemographic and occupational characteristics. In the second part, the dermatologists were asked which dermatological, surgical, and cosmetic procedures they performed and how often. The third part asked whether they experienced emergency complications during these procedures. Emergency complications were classified as vasovagal syncope, seizure, anaphylaxis, hyperventilation syndrome, hypotension/bleeding, and cardiac arrest. The last part inquired about the dermatologists' level of knowledge regarding emergency complications and basic life support.

The data analysis was conducted with SPSS Statistics for Windows version 21.0 (IBM Corp., Armonk, NY). Continuous data are expressed as mean ± standard deviation for normally distributed data and as median, 25th percentile, and 75th percentile for non‐normally distributed data. Categorical data are given as percentages (%). The Shapiro–Wilk test was used to determine whether the data were normally distributed. In comparing groups that did not show normal distribution, the Mann–Whitney *U*‐test was used when the number of groups was two, whereas one‐way analysis of variance was used to compare groups of three or more. Tukey's multiple comparison test was used for multiple comparisons of groups with significant differences, and Pearson's exact chi‐squared analysis was used in the analysis of the cross tables. The results were evaluated within the 95% confidence interval and were considered statistically significant when the alpha error was less than 5% (*p* < 0.05).

## 3 RESULTS

A total of 240 dermatologists participated in the study. Their mean age was 40.27 years, and 81% were female. The sociodemographic and occupational characteristics of the dermatologists are summarized in Tables 1 and 2.

**TABLE 1:** Sociodemographic characteristics of the dermatologists.

	*n* (%)
Sex	
Female	195 (81)
Male	45 (19)
Age (year)	
Mean ± standard deviation (min–max)	40.27 ± 10.86 (15–67)
Academic degree	
Resident	55 (23)
Assistant professor	21 (9)
Associate professor	17 (7)
Professor	17 (7)
Specialist	130 (54)
Professional experience (years)	
0–4	55 (23)
5–10	37 (15)
10–15	37 (15)
>15	111 (47)
Institution of residency training	
State university hospital	146 (61)
Foundation university hospital	4 (2)
Training and research hospital	87 (36)
Abroad	3 (1)
Current institution	
Private clinic	64 (27)
University hospital	62 (26)
Training and research hospital	45 (19)
Private hospital	36 (15)
Public hospital	24 (10)
Medical center	9 (4)

**TABLE 2:** Occupational characteristics of dermatologists.

	*n* (%)
Average number of dermatological/surgical procedures per week	
<10	68 (28)
10–50	115 (49)
50–100	37 (15)
>100	20 (8)
Dermatological/surgical procedure	
Cryotherapy	222 (93)
Biopsy	232 (97)
Excision	157 (65)
Electrocauterization	229 (95)
Intralesional injection	239 (99)
Nail surgery	124 (52)
Skin grafting	9 (4)
Performing cosmetic procedures	
Yes	194 (81)
No	46 (19)
Average number of cosmetic procedures per week	
<10	100 (51.5)
10–50	71 (36.6)
50–100	16 (8.2)
>100	7 (3.7)
Botulinum toxin	180 (75)
Platelet‐rich plasma	178 (74)
Mesotherapy	175 (73)
Micro‐needling	130 (54)
Dermal filler	116 (48)
Chemical peeling	105 (44)
Thread	52 (22)
Laser	112 (47)
Radiofrequency	89 (37)
Stem cell therapy	43 (18)
Hair transplantation	10 (4)

Emergency complications during dermatological and surgical procedures were reported by 151 (53%) dermatologists, with the most commonly reported complications being vasovagal syncope (58%), hypotension/bleeding (28%), and seizures (10%). Emergency complications were most common during biopsies (34%) and intralesional injections (27%). The most common anatomical localization of emergency complications was the head and neck region (50%) (Table 3).

**TABLE 3:** Emergency complications during dermatological/surgical and cosmetic procedures.

	*n* (%)
Dermatological/surgical procedures	
Emergency complications	
Yes	151 (63)
No	89 (37)
Type of emergency complication	
Vasovagal syncope	140 (58)
Seizure	25 (10)
Anaphylaxis	7 (3)
Hyperventilation syndrome	7 (3)
Hypotension/bleeding	68 (28)
Cardiac arrest	2 (0.8)
Anatomical localization	
Head and neck	120 (50)
Body	28 (12)
Extremities	44 (18)
Nail	16 (7)
Type of dermatological/surgical procedure	
Cryotherapy	28 (12)
Biopsy	82 (34)
Excision	28 (12)
Electrocauterization	14 (6)
Intralesional injection	65 (27)
Nail surgery	19 (8)
Skin grafting	2 (0.8)
Cosmetic procedures	
Emergency complications	
Yes	84 (43.2)
No	110 (56.8)
Type of emergency complication	
Vasovagal syncope	75 (38.6)
Seizure	11 (5.6)
Anaphylaxis	6 (3)
Hyperventilation syndrome	6 (3)
Hypotension/bleeding	27 (13.9)
Cardiac arrest	1 (0.5)
Type of cosmetic procedure	
Botulinum toxin	33 (17)
Platelet‐rich plasma	18 (9.3)
Mesotherapy	31 (15.9)
Micro‐needling	7 (3.6)
Dermal filler	46 (23.7)
Chemical peeling	1 (0.5)
Thread	13 (7.2)
Laser	7 (3.6)
Radiofrequency	3 (1.5)
Stem cell therapy	5 (2.5)
Hair transplantation	8 (4.12)

Of the 194 dermatologists who performed cosmetic procedures, 84 (43.2%) reported emergency complications. The most common reported complications were vasovagal syncope (38.6%), hypotension/bleeding (13.9%), and seizures (5.6%). Emergency complications were most common during injections of dermal filler (23.7%) and botulinum toxin (17%) (Table 3).

We asked the dermatologists whether they had previously undergone basic life support training; 65 (27%) reported having no training, while 74 (31%) had only theoretical training, and 101 (42%) had both theoretical and practical training.

We also evaluated the relationship between emergency complications and the dermatologists' sociodemographic and occupational characteristics. Emergency complications were significantly more common among specialists, those with more than 15 years of professional experience, those working in their private clinics, those with an average of 10–50 dermatological/surgical procedures per week, and those with an average of fewer than 10 cosmetic procedures per week (*p* < 0.05) (Table 4).

**TABLE 4:** Relationship between emergency complications and sociodemographic and occupational characteristics.

	Emergency complications during dermatological/surgical and cosmetic procedures		*p* ^a^
	Yes *n* (%)	No *n* (%)	
Sex			
Male	31 (19.5)	14 (17.28)	0.81
Female	128 (80.5)	67 (82.72)	
Academic degree			
Resident	21 (13.21)	34 (41.98)	<0.001
Assistant professor	15 (9.43)	6 (7.41)	
Associate professor	16 (10.06)	1 (1.23)	
Professor	13 (8.18)	4 (4.94)	
Specialist	94 (59.12)	36 (44.4)	
Professional experience (years)			
0–4	21 (13.21)	34 (41.98)	<0.001
5–10	24 (15.09)	13 (16.05)	
10–15	26 (16.35)	11 (13.58)	
>15	88 (55.35)	23 (28.4)	
Institution of residency training			
State university hospital	96 (60.38)	50 (61.73)	0.64
Foundation university hospital	3 (1.89)	1 (1.23)	
Training and research hospital	57 (35.85)	30 (37.04)	
Abroad	3 (1.89)	0 (0)	
Current institution			
Private clinic	51 (32.08)	13 (16.05)	0.01
University hospital	36 (22.64)	26 (32.1)	
Training and research hospital	23 (14.47)	22 (27.16)	
Private hospital	28 (17.61)	8 (9.88)	
Public hospital	14 (8.81)	10 (12.35)	
Medical center	7 (4.4)	2 (2.47)	
Average number of dermatological/surgical procedures per week			
<10	46 (28.93)	22 (27.16)	0.03
10–50	83 (52.2)	32 (39.5)	
50–100	22 (13.84)	15 (18.52)	
>100	8 (5.03)	12 (14.81)	
Average number of cosmetic procedures per week			
<10	60 (45.1)	40 (65.6)	0.028
10–50	53 (39.8)	18 (29.5)	
50–100	13 (9.8)	3 (4.9)	
>100	7 (5.3)	0 (0)	
Would you like to have a basic life support course?			
Yes	149 (93.71)	78 (96.30)	0.59
No	10 (6.29)	(3.70)	

^a^ Pearson's exact chi‐squared test.

The participating dermatologists were asked to evaluate their knowledge/skill level regarding emergency complications; 112 (47%) described their knowledge/skill level as fair, 73 (30%) as poor, 4 (2%) as very poor, 43 (18%) as good, and 8 (3%) as very good. The next part of the questionnaire evaluated the dermatologists' knowledge of emergency complications and basic life support as summarized in Table 5.

**TABLE 5:** Knowledge level of dermatologist on emergency complications and basic life support.

Question	Correct answer *n* (%)	Wrong answer *n* (%)
What is the first and most important drug in the treatment of anaphylaxis?	237 (99)	3 (1)
Which method do you prefer when applying adrenaline in the treatment of anaphylaxis?	129 (54)	111 (46)
What is the dose of adrenaline used in the treatment of anaphylaxis?	141 (59)	99 (41)
In anaphylaxis, when it is necessary to reapply adrenaline according to the adrenaline response, after how many minutes should it be administered?	116 (48)	124 (52)
Which position would you prefer to give to the unconscious patient who has breathing and circulation?	62 (26)	178 (74)
What can be done to prevent vasovagal syncope?	98 (41)	142 (59)
Which drug is used to treat vasovagal syncope?	108 (45)	132 (55)
How should a patient with hyperventilation syndrome be treated?	121 (50.5)	119 (49.5)
In what order is it recommended to perform the basic life support steps in an unconscious patient?	121 (50.5)	119 (49.5)
What should be the compression/ventilation ratio according to basic life support steps in a patient with cardiac arrest?	119 (49.5)	121 (50.5)
What should be the compression rate according to basic life support steps in a patient with cardiac arrest?	99 (41)	141 (59)
How often should pulse be controlled according to basic life support steps in a patient with cardiac arrest?	173 (72)	67 (28)

We evaluated the relationship between the dermatologists' knowledge level regarding emergency complications and basic life support and their sociodemographic and occupational characteristics. The knowledge scores were highest among residents, dermatologists with 0–4 years of experience, dermatologists working in university hospitals, and dermatologists with both theoretical and practical training in basic life support (Table 6).

**TABLE 6:** Relationship between knowledge level of dermatologist on emergency complications and basic life support and sociodemographic and occupational characteristic.

	Total score	*p* ^a^	Multiple comparison *p* ^b^
Academic degree			
Resident (1)	8.13 ± 2.11	<0.001	1–5: <0.001
Specialist (2)	5.55 ± 2.34		1–2: <0.001
Assistant professor (3)	7.76 ± 2.12		3–5: <0.001
Associate professor (4)	6.41 ± 2.53		2–3: <0.001
Professor (5)	4.94 ± 2.11		1–2: 0.0049
Professional experience (years)			
0–4 (1)	7.95 ± 2.21	<0.001	
5–10 (2)	7.03 ± 2.53		1–3: 0.01
10–15 (3)	6.43 ± 2.36		1‐4: <0.001
>15 (4)	5.31 ± 2.28		2–4: <0.001
Institution of residency training			
Training and research hospital	6.38 ± 2.40	0.160	‐
State university hospital	6.42 ± 2.63		
Foundation university hospital	5.25 ± 1.89		
Abroad	3.33 ± 1.53		
Current institution			
Private clinic (1)	4.94 ± 2.08	<0.001	
University hospital (2)	7.73 ± 2.21		1–3: <0.001
Training and research hospital (3)	6.96 ± 2.59		1–2: <0.001
Private hospital (4)	6.00 ± 2.55		2–4: <0.001
Public hospital (5)	6.25 ± 2.52		
Medical center (6)	5.59 ± 1.94		
Have you had a basic life support course?			
No (1)	5.65 ± 2.31	<0.001	
Theoretical training (2)	5.95 ± 2.50		1–3: <0.001
Theoretical and practical training (3)	7.10 ± 2.54		2–3: <0.001
Would you like to have a basic life support course?			
Yes	6.32 ± 2.52	0.47	‐
No	6.385 ± 2.97		

^a^ One‐way analysis of variance.

^b^ Tukey's multiple comparison test.

Finally, the dermatologists were asked whether they would like to have training programs/courses on emergency complications and basic life support; 227 (95%) indicated that they would like to participate in such courses.

## 4 DISCUSSION

In this study, 53% of dermatologists reported emergency complications during dermatological and surgical procedures and 43.2% during cosmetic procedures. Complication rates were higher in dermatological and surgical procedures than in cosmetic ones, perhaps because patients are more motivated in cosmetic procedures and are more concerned about the aesthetic outcome than about potential damage. This may explain why some complications, such as vasovagal syncope, are less common in cosmetic procedures (38.6%) than in dermatological/surgical ones (58%). In addition, patients undergoing cosmetic procedures are generally healthy, whereas those undergoing dermatological and surgical procedures may have medical comorbidities that could lead to more frequent emergency complications. In a study in the United States, Hazen et al. examined the incidence of medical emergencies in the practices of 34 dermatologists, finding 55 emergency events over 565 physician‐years (one episode every 10.3 years). They reported that emergencies were more frequent in medical procedures than in surgical ones.^5^

We also assessed the relationship between emergency complications and the dermatologists' sociodemographic and occupational characteristics. Emergency complications were more commonly reported among specialists, those with more than 15 years of professional experience, those working in their private clinics, and those with an average of 10–50 dermatological/surgical procedures per week, and those with an average of fewer than 10 cosmetic procedures per week. Similarly, Hazen et al. indicate that all the emergency events were reported by those with over 5 years of professional experience.^5^ More experienced and private‐practice dermatologists may have been more willing to participate in the study and have been more confident in reporting complications, as they have performed more procedures over the years and experienced more complications. Conversely, less experienced dermatologists may have reported fewer complications because of fear of being judged, even though they were told that the participants' identities would be anonymous to the researchers in this study. We would expect more complications among dermatologists who performed more cosmetic procedures, but dermatologists who performed fewer procedures reported more complications in our study.

In our study, the most commonly reported complications were vasovagal syncope, hypotension/bleeding, and seizures. In a French study, Amici et al. prospectively evaluated the incidence of anesthetic, hemorrhagic, and infectious complications in the dermatological surgery practices of 84 dermatologists for 3 months. They found only 236 complications in a total of 3788 surgical procedures (6%), a lower rate of complications than in our study.^2^ In another prospective study, the incidence of complications in Mohs micrographic surgery was found to be 1.6%.^3^ The difference in the results of these studies may be due to differences in methodology. Hazen et al. found 55 emergency events over 565 physician‐years (1 episode every 10.3 years). Much as in our study, the most common emergencies were vasovagal episodes (15 events), cardiac emergencies (13 events, 3 of them cardiac arrest), and seizures (5 events).^5^

Vasovagal syncope, one of the more common emergency complications in dermatological practice, is caused by a disruption of the adaptive mechanisms that maintain normal blood pressure and cerebral blood supply in susceptible individuals. Loss of sympathetic tone causes hypotension, intense vagal stimulation causes bradycardia, and syncope develops as a result of cerebral hypoperfusion. Standing for a prolonged time, emotional stress, fasting, a hot environment, fear, and pain can trigger vasovagal syncope, and common symptoms include dizziness, pallor, sweating, and nausea.^6^, ^7^, ^8^, ^9^, ^10^ Although it may alarm physicians, vasovagal syncope is generally a benign, transient, and self‐resolving condition, but it can cause cardiac arrest in some patients. It was the most common emergency complication in our study, reported by 58% of participants during dermatological/surgical procedures and 38.6% during cosmetic procedures. Amici et al. report a lower rate of vasovagal syncope than in our study; the incidence of vasovagal syncope was 1% in their cohort (6% of it before surgery, 45% during surgery, 37% after surgery, and 12% unknown).^2^

To prevent vasovagal syncope, physicians should advise their patients to take enough liquid and food before the procedure and avoid alcohol intake. Patients should not be kept standing for a long time before the procedure, and procedures should be performed with patients in the supine position. Stimuli such as blood and needles should be removed from the patient's field of vision. Isometric counter‐pressure maneuvers (leg crossing, pulling hands in opposite directions, squeezing a rubber ball) may also prevent vasovagal syncope. In the management of patients with vasovagal syncope, they should first be placed in the Trendelenburg position to facilitate brain perfusion. Subcutaneous injection of 1 mL of 0.4 mg/mL atropine, a muscarinic acetylcholine receptor antagonist, should be considered to reverse vagal cardiac stimulation.^7^, ^9^ We asked the dermatologists which position patients with vasovagal syncope should assume, what can be done to prevent vasovagal syncope, and which drug is used to treat the condition. Most gave the correct answers (74%, 59%, and 55%, respectively).

The second most common emergency complication in our study was hypotension/bleeding, reported by 28% of dermatologists performing dermatological/surgical procedures and 13.9% of those performing cosmetic procedures. Amici et al. report hemorrhagia in 103 procedures (3%) and detected it more commonly in males; when immunosuppressant, anticoagulant, or antiplatelet drugs were used; and in prolonged procedures, skin flaps, and skin grafts.^2^ Bordeaux et al. report hemorrhagic complications in 0.89% of 1911 patients who underwent dermatological surgery. None of the patients were hospitalized, and long‐term sequelae were not observed.^4^

Seizures and status epilepticus are believed to be encountered infrequently in dermatology practice,^11^ yet in our study seizures were reported by 28% of dermatologists performing dermatological/surgical procedures and 5.6% of those performing cosmetic procedures. Hazen et al. report that seizures were the third most common emergency in dermatological practice.^5^ In a study investigating the prevalence of medical emergencies in dental offices in Poland, participants reported seizures in 11.81% of procedures.^1^ Seizures require immediate intervention with basic life support steps and trauma prevention. If the seizure lasts longer than 10 min, lorazepam may be administered.^11^

Anaphylaxis is a severe systemic reaction with sudden onset that can be fatal. Symptoms usually start on the skin as pruritus, erythema, or urticaria and proceed to bronchospasm, hypotension, and arrhythmias.^7^, ^8^, ^12^ The incidence of anaphylaxis in dermatology offices is not well known but is believed to be low. In our study, anaphylaxis was reported by 3% of the dermatologists during dermatological, surgical, or cosmetic procedures. In dermatology offices, anaphylaxis may result from presurgical prophylactic antibiotic use, local anesthetic injection, contact with latex, and (rarely) topical application of antibiotics or disinfectants.

Prompt diagnosis is crucial in managing anaphylaxis. The inciting agent should be removed if detected. Basic life support steps (related to the airway, breathing, and circulation) should be performed, and intramuscular injection of 0.01 mg/kg of 1:1000 adrenaline solution is the first‐line treatment; the dose can be repeated every 5–15 min. Referral to emergency medical services or intensive care units should be planned.^7^, ^12^ We asked the dermatologists about managing anaphylaxis, and almost all (99%) knew that adrenaline was the treatment of first choice, but only about half of them gave correct answers to the other questions on anaphylaxis (route of adrenaline administration, dose of adrenaline, time for reapplying adrenaline).

Hyperventilation syndrome, an anxiety‐related increase in breathing resulting in decreased CO_2_ pressure in the blood and cerebral vasoconstriction, is characterized by anxiety, shortness of breath, nausea, perioral numbness, paresthesia, and tetany of the hands. Prolonged hyperventilation syndrome may result in syncope. In its management, the patient should breathe abdominally and superficially.^7^ In our study, 3% of dermatologists reported hyperventilation syndrome, and approximately half gave the correct answer when asked how a patient with hyperventilation syndrome should be treated.

Cardiac arrest was reported in our study by 0.8% of the dermatologists during dermatological/surgical procedures and by 0.5% during cosmetic procedures. Although the condition has been reported in very few patients, it is important, as it can result in death, so dermatologists should be prepared for the risk of cardiac arrest during daily practice. Management of cardiac arrest is based on cardiopulmonary resuscitation and defibrillation in the presence of ventricular fibrillation or ventricular tachycardia. Patient survival depends on basic life support, advanced cardiovascular life support, and post–cardiac arrest care.^12^

We asked the dermatologists about basic life support and cardiopulmonary resuscitation, and approximately half gave correct answers. When we asked them to describe their knowledge/skill levels regarding emergency complications, 47% indicated that their knowledge/skill level was adequate, 30% poor, 2% very poor, 18% good, and 3% very good. A large proportion of dermatologists (95%) reported that they would like to participate in training programs/courses on emergency complications and basic life support. The knowledge level of dermatologists was highest among residents, dermatologists with 0–4 years of professional experience, those working in university hospitals, and those who had both theoretical and practical training in basic life support. These findings have two important implications. First, because basic life support training was given in medical school, the knowledge level of residents and young dermatologists may have been better and more up to date. This suggests that it may be beneficial to repeat training programs on basic life support and emergencies at regular intervals after graduation. Additionally, dermatologists with theoretical and practical training had higher knowledge levels, so both theoretical and practical training should be provided.

To the best of our knowledge, this is the first study evaluating the frequency of dermatologists who experienced emergency complications during dermatological, surgical, or cosmetic procedures and evaluating their level of knowledge on basic life support. However, our study has some limitations. First, we could not evaluate patient characteristics due to our study's cross‐sectional design. Second, this study was conducted online, and dermatologists who experienced dermatological complications more frequently or who were more interested in this issue may have been overrepresented, resulting in bias. Third, the emergency complications were only recorded in dermatologists' memories or claims rather than being formally documented. Finally, the questionnaire asked dermatologists whether they had experienced certain complications but not how many times they had experienced them, as the participants were asked to make an evaluation that covered their entire professional practice period. Because the study was based on self‐reported statements, it was considered that asking for the number of complications would not yield an accurate result.

## 5 CONCLUSION

This study shows a relatively high frequency of dermatologists who experienced emergency complications during dermatological, surgical, or cosmetic procedures. Although these complications appear to be common, most of them are mild, self‐limiting, and not life threatening, suggesting the safety of these procedures on an outpatient basis. Nevertheless, dermatologists should be competent and prepared to intervene in medical emergencies in daily practice. Theoretical and practical training programs on basic life support and emergencies at regular intervals after graduation should be provided for dermatologists.

## AUTHOR CONTRIBUTIONS

H.K.E., M.S.T., E.A., and S.S.B. designed the research study, H.K.E., M.S.T., E.A., and E.Ac. performed the research, H.K.E., M.S.T., Z.N.S., and M.B. analyzed the data. H.K.E. wrote the paper.

## ETHICS STATEMENT

The Eskişehir Osmangazi University Ethics Committee approved the study protocol (date: 26.7.2022, number: 3).

## Data Availability

The data that support the findings of this study are available on request from the corresponding author. The data are not publicly available due to privacy or ethical restrictions.
